# Hypotension and cognitive impairment among the elderly: Evidence from the CLHLS

**DOI:** 10.1371/journal.pone.0291775

**Published:** 2023-09-19

**Authors:** Xidi Zhu, Zhicheng Luo, Gang Tian, Zhao Hu, Shaojie Li, Qing Mei Wang, Xun Luo, Lizhang Chen

**Affiliations:** 1 Department of Epidemiology and Health Statistics, Xiangya School of Public Health, Central South University, Changsha, China; 2 Stroke Biological Recovery Laboratory, Spaulding Rehabilitation Hospital, the Teaching Affiliate of Harvard Medical School, Boston, Massachusetts, United States of America; 3 Department of Social Medicine and Health Management, Xiangya School of Public Health, Central South University, Changsha, China; 4 School of Public Health, Peking University, Beijing, China; 5 Kerry Rehabilitation Medicine Research Institute, Shenzhen, China; 6 School of Medicine, Shenzhen University, Shenzhen, China; Ehime University Graduate School of Medicine, JAPAN

## Abstract

**Background:**

While high blood pressure has been linked to cognitive impairment, the relationship between low blood pressure, especially hypotension, and cognitive impairment has not been well studied. Therefore, this study aimed to assess the prevalence of hypotension and cognitive impairment in the seniors of China, and the association between hypotension and cognitive function impairment.

**Methods:**

The data was derived from the 2018 wave of the Chinese Longitudinal Healthy Longevity Survey (CLHLS). Systolic blood pressures (SBP) and diastolic blood pressures (DBP) were measured by objective examination. The Chinese version of the Mini-Mental State Examination (CMMSE) was used to evaluate the cognitive impairment of the elderly. Generalized linear models were conducted to evaluate the association of hypotension with cognitive impairment.

**Results:**

The prevalence of hypotension and cognitive impairment in the Chinese elderly were 0.76% and 22.06%, respectively. Participants with hypotension, lower SBP, and lower DBP, had odds ratios of 1.62, 1.38, and 1.48 for cognitive impairment, respectively. Besides, the CMMSE scores decreased by 2.08, 0.86, and 1.08 in the elderly with hypotension, lower SBP, and DBP, compared with those with non-hypotension, higher SBP, and DBP, respectively. Subgroup analyses showed that the association of cognitive impairment with hypotension was stronger in Chinese elderly who had decreased activity of daily living. Moreover, there was statistical evidence of a nonlinear dose-response relationship of SBP and DBP with cognitive impairment (*P*_nonlinear_ < 0.05).

**Conclusion:**

Hypotension was a potential risk factor for cognitive impairment of the Chinese elderly, especially for those having decreased activity of daily living. Blood pressure management should be conducted to prevent them from cognitive impairment.

## Introduction

Aging has become a serious problem at the global level, and it is estimated that half of the additional 1 billion people added to Asia’s population by 2050 will be over the age of 65 [1]. In China, the proportion of elderly people among its total population will be 22.6% by 2040 [2]. Therefore, it is of great importance to care for the health of the elderly. Blood pressure is one of the most fundamental cardiovascular concerns in the elderly, which may have a significant impact on their cognitive function [3].

Previous studies have suggested that blood pressure is strongly related to cognitive impairment in the elderly. Evidence has shown significant associations between high blood pressure and dementia, cognitive function decline, and Alzheimer’s Disease (AD) in older adults [4–7]. In terms of hypotension, there were inconsistent findings. For example, a prospective national study from the USA indicated that faster declines in global cognition were significantly associated with higher systolic blood pressure (SBP) and lower diastolic blood pressure (DBP) with increasing age [8]. A cross-sectional study in China and several longitudinal studies in the United States and Sweden reported a significant linear relationship between greater declines in blood pressure and cognitive function impairment or senile dementia in people over 65 years of age [9–12]. Others reported mixed results. For instance, a retrospective cohort study in the Netherlands showed that hypotensive syndromes were not associated with cognitive impairment in geriatric patients [13]. Based on a multicenter research study in Italy, Zuccalà et al. found that systolic hypotension was associated with cognitive impairment only in those elderly with heart failure and not in those without heart failure [14].

Identifying risk factors for cognitive impairment and taking targeted preventive measures for the elderly were effective ways to delay the onset of cognitive disorders, especially when treatment for cognitive impairment was limited [15, 16]. Given that hypotension is a potential risk factor contributing to the development of cognitive impairment, assessing the association between hypotension and cognitive impairment in the Chinese elderly might provide scientific evidence for policy-making to prevent cognitive impairment. Therefore, in this study, we aimed to investigate 1) the prevalence of hypotension and cognitive impairment in the seniors of China, and 2) the association between hypotension and cognitive function impairment in older people, based on the Chinese Longitudinal Healthy Longevity Survey (CLHLS) that was a representative nationwide investigation.

## Materials and methods

### Data source

The data for the current study was derived from the 2018 wave of the Chinese Longitudinal Healthy Longevity Survey (CLHLS). Briefly, the CLHLS was a nationally representative investigation of the elderly aged over 65 years conducted by the Center for Healthy Aging and Development Studies of Peking University using the probability proportionate to size sampling method. The investigation began in 1998, and follow-up surveys were conducted in 2000, 2002, 2005, 2008, 2011, 2014, and 2018. Informed consent was obtained from all participants, and then all of them received a face-to-face interview to finish a questionnaire that included health status, family information, and so on. The details of the CLHLS were presented online [17]. The ethics review was approved by the Ethics Committee of Peking University (approval number: IRB00001052-13074). All the participants gave their written informed consent at his or her enrollment. In this study, we excluded participants aged under 65 years (103) as well as those with missing blood pressure measurement (192), CMMSE information (31), and those with anemia (11). Finally, our study included 15,537 participants in its analysis.

### Dependent variables

#### Cognitive impairment

Cognitive function of the seniors was measured by the Chinese version of the Mini-Mental State Examination (CMMSE), which was appropriate for the seniors with Chinese cultural backgrounds in CLHLS. The scale contains 24 items in 6 dimensions with a total score ranging from 0 to 30 points: 5 items for Orientation, 3 for Registration, 1 for Naming, 5 for Attention and Calculation, 3 for Recall, and 7 for Language. Higher scores indicate better cognitive function. The CMMSE has been used in previous studies and shown to be validated when compared to other versions of the MMSE [18]. A CMMSE score of fewer than 18 points signifies cognitive impairment, and a score of 2 represents the minimal clinically important difference (MCID) of CMMSE [19].

### Independent variables

#### Hypotension

During the investigation, the participants were asked to measure their blood pressure twice at home before the meal and antihypertensive medication, operated by the trained research assistants. Generally, the subject was seated in a chair with feet flat on the floor and arms resting on a table, and specifically, bedridden subjects had their blood pressure measured in a supine position. The mercurial sphygmomanometer (upper arm type; Yuyue, Jiangsu, China) was at the heart level of the subjects, and must be calibrated again before each blood pressure measurement. A more detailed description of blood pressure measurements is shown in S1 File. According to two objective blood pressure measurements taken at least one minute apart, SBP and DBP were calculated as the average of the two measurements taken for an individual. The Pearson correlation coefficients between two blood pressure measurements were 0.910 (*P* <0.001) for two SBP measurements and 0.794 (*P* <0.001) for two DBP measurements, indicating significant correlations between two objective blood pressure measurements [20]. In accordance with the Chinese criteria, hypotension was defined as SBP ≤ 98 mm Hg and DBP ≤ 60 mm Hg [21, 22]. Furthermore, lower SBP was defined as SBP ≤ 98 mm Hg, and lower DBP was defined as DBP ≤ 60 mm Hg. Therefore, in statistics analysis, SBP and DBP were also considered as independent variables, where SBP was categorized into two groups ≤ 98 mm Hg and > 98 mm Hg, and DBP was divided into ≤ 60 mm Hg and > 60 mm Hg. To further test the robustness of the association between hypotension and cognitive impairment, we classified hypotension as a multicategory variable: no-hypotension (SBP > 98 mm Hg and DBP > 60 mm Hg), isolated systolic hypotension (SBP ≤ 98 mm Hg and DBP > 60 mm Hg), isolated diastolic hypotension (SBP > 98 mm Hg and DBP ≤ 60 mm Hg), and sustained hypotension (SBP ≤ 98 mm Hg and DBP ≤ 60 mm Hg).

### Covariates

Potential confounding factors were selected based on experience and reports from previous studies [23, 24]. Generally, confounders involve demographics characteristics, lifestyle habits, health status, history of substance use, and other related factors. Demographic characteristics, lifestyle, and health status were considered as covariates. Demographic characteristics included gender (male/female), age (65–80 or >80 years), education level (≤0 or >0 years), marital status (married or other), living arrangement (with family members or without family members), and registered residence (urban or rural). Smoking at present (yes or no), drinking alcohol at present (yes or no), and exercising at present (yes or no) reflected the lifestyle of the seniors. Health status involved body mass index (BMI) (normal or abnormal), hypertension (yes or no), heart diseases (yes or no), senile dementia (yes or no), Parkinson’s disease (yes or no), diabetes (yes or no), stroke (yes or no), and functional disability (damaged or undamaged). Specifically, a normal BMI was defined as 18.5 to 24.9, otherwise, it was abnormal. Participants who self-reported being diagnosed with hypertension by II&III grade hospitals before were considered to have a history of hypertension. Similarly, personal history of diabetes, heart diseases, stroke, senile dementia, and Parkinson’s diseases were also based on self-reported. Function disability was measured by the basic activities of daily living (ADL) scale that consists of the following six items: bathing, dressing, toileting, indoor transferring, eating, and continence. Each option has three answers: receives no assistance, receives assistance only for part of the body, and receives assistance more than one part of the body, with corresponding scores of 1, 2, and 3, respectively. The function in basic ADL was categorized as damaged (total scores > 6) and undamaged (total scores ≤ 6). History of substance use referred to the use of antihypertensive and hypoglycemic medications within 24 hours and was collected based on the individual’s self-reporting.

### Statistics analysis

Descriptive analyses were presented as counts and proportions for categorical variables, and Chi-square tests were used to compare the differences between groups. We used generalized linear models to evaluate the odds ratios (ORs) and corresponding 95% confidence intervals (CIs) of the associations of hypotension, SBP, and DBP with cognitive impairment. Additionally, generalized linear models were applied to assess the βs and 95% CIs of CMMSE scores with hypotension, SBP, and DBP. Before multivariate analysis, we first selected the independent variables that were statistically significant in the crude model. Then we tested the collinearity of these variables and excluded those with high collinearity. Finally, we included all eligible independent variables in the final model. In the multivariable-adjusted model, the following independent variables were selected: age, sex, education level, marital status, smoking status, drinking status, exercise, BMI, living arrangement, cardiovascular disease, diabetes, hypertension, functional disability, stroke, senile dementia, Parkinson’s diseases, registered residence, antihypertensive medication, and hypoglycemic agents. We used restricted cubic splines (RCS) to model the dose-response relationship among SBP, DBP, and cognitive impairment [25]. Moreover, we performed subgroup analyses by the former independent variables. To test effect modification of selected factors, we added a cross-product term into separate models to assess the significance of interaction terms. Additionally, we conducted sensitivity analysis using 24 (≤23) as the CMMSE cut-off point to evaluate the robustness of the association between hypotension and cognitive impairment [26]. *P*-values < 0.05 were considered statistically significant, except for the interaction analyses where *P*-values < 0.10 were used.

## Results

**Fig 1:**
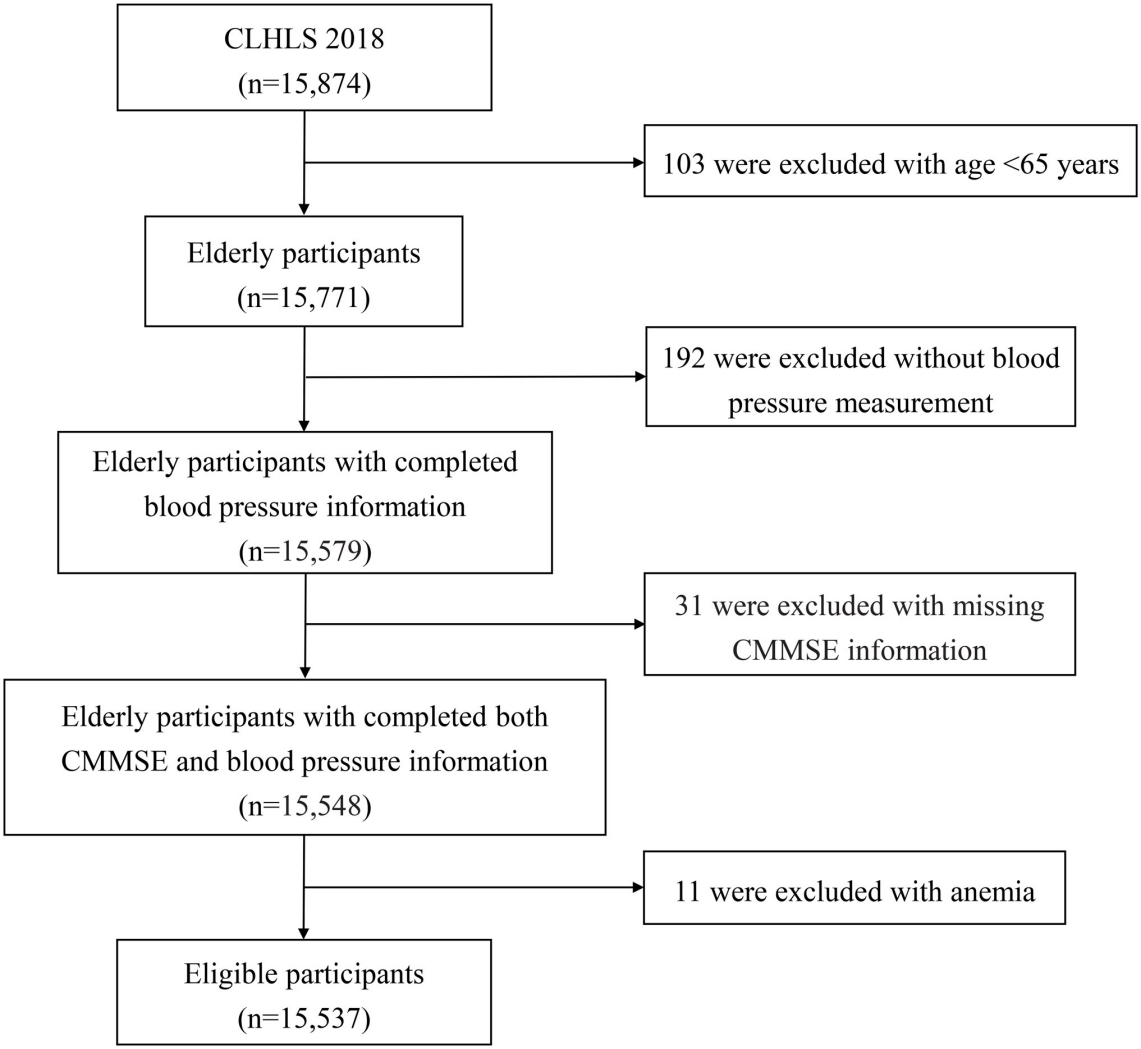
Flow chart of eligible participants included in the analysis.

This study included 15,537 seniors from the 2018 wave of the CLHLS (Fig 1). The prevalence of hypotension and cognitive impairment in the Chinese elderly were 0.76% and 22.06%, respectively. Besides, the prevalence of cognitive impairment was higher in women than in men (27.95% *vs*.14.46%). Hypotension was more likely to be suffered in participants who were over 80 years of age, unmarried, currently smoking, and in those with irregular exercise, abnormal BMI, functional disability, and senile dementia. When compared to participants without cognitive impairment, participants with cognitive impairment showed significant differences in all sociodemographic and health-related variables except BMI. Basic characteristics of the study participants are represented in Table 1.

**Table 1:** Descriptive characteristics of study population according to hypotension and cognitive function status in the 2018 survey wave.

Characteristics	Hypotension, n (%)		*p*	Cognitive impairment, n (%)		*p*
	Yes	No		Yes	No	
Gender			0.302			<0.001
Male	46 (39.0)	6740 (43.7)		981 (28.6)	5805 (47.9)	
Female	72 (61.0)	8679 (56.3)		2446 (71.4)	6305 (52.1)	
Age (years)			<0.001			<0.001
65–80	22 (18.6)	5727 (37.1)		147 (4.3)	5602 (46.3)	
>80	96 (81.4)	9692 (62.9)		3280 (95.7)	6508 (53.7)	
Education level (years)			0.125			<0.001
0	62 (60.2)	6656 (50.2)		2340 (77.8)	4378 (42.2)	
1~6	25 (24.3)	4139 (31.2)		461 (15.3)	3703 (35.7)	
>6	16 (15.5)	2475 (18.7)		205 (6.8)	2286 (22.1)	
Marital status			0.001			<0.001
Married	28 (23.9)	6013 (39.4)		382 (11.3)	5659 (47.2)	
Others	89 (76.1)	9252 (60.6)		3008 (88.7)	6333 (52.8)	
Living arrangement			0.034			<0.001
Alone	10 (8.5)	2435 (16.0)		395 (11.7)	2050 (17.2)	
Nursing home	7 (6.0)	558 (3.7)		220 (6.5)	345 (2.9)	
Household	100 (85.5)	12200 (80.3)		2770 (81.8)	9530 (79.9)	
Smoking status			0.009			<0.001
Yes	27 (23.3)	2237 (14.7)		264 (7.8)	2000 (16.7)	
No	89 (76.7)	13020 (85.3)		3129 (92.2)	9980 (83.3)	
Drinking status			0.227			<0.001
Yes	12 (10.3)	2148 (14.2)		255 (7.6)	1905 (16.0)	
No	105 (89.7)	13021 (85.8)		3108 (92.4)	10018 (84.0)	
Exercise			0.019			<0.001
Yes	23 (20.0)	4567 (30.1)		337 (10.0)	4253 (35.7)	
No	92 (80.0)	10621 (69.9)		3037 (90.0)	7676 (64.3)	
Functional disability			<0.001			<0.001
Damaged	57 (48.3)	3998 (26.0)		2261 (66.0)	1794 (14.8)	
Undamaged	61 (51.7)	11406 (74.0)		1164 (34.0)	10303 (85.2)	
BMI			0.002			<0.001
Normal	39 (33.1)	7279 (47.2)		1334 (38.9)	5984 (49.4)	
Abnormal	79(66.9)	8140 (52.8)		2093 (61.1)	6126 (50.6)	
Hypertension			<0.001			<0.001
Yes	25 (21.2)	6362 (41.3)		1044 (30.5)	5343 (44.1)	
No	93 (78.8)	9057 (58.7)		2383 (69.5)	6767 (55.9)	
Diabetes			0.393			<0.001
Yes	8 (6.8)	1394 (9.0)		164 (4.8)	1238 (10.2)	
No	110 (93.2)	14025 (91.0)		3263 (95.2)	10872 (89.8)	
Cardiovascular diseases			0.804			<0.001
Yes	18 (15.3)	2482 (16.1)		440 (12.8)	2060 (17.0)	
No	10 (84.7)	12937 (83.9)		2987 (87.2)	10050 (83.0)	
Stroke			0.639			0.039
Yes	14 (11.9)	1624 (10.5)		394 (11.5)	1244 (10.3)	
No	104 (88.1)	13795 (89.5)		3033 (88.5)	10866 (89.7)	
Senile dementia			0.051			<0.001
Yes	6 (5.1)	345 (2.2)		299 (8.7)	52 (0.4)	
No	112 (94.9)	15074 (97.8)		3128 (91.3)	12058 (99.6)	
Parkinson’s diseases			0.240			0.001
Yes	2 (1.7)	121 (0.8)		42 (1.2)	81 (0.7)	
No	116 (98.3)	15298 (99.2)		3385 (98.8)	12029 (99.3)	
Registered residence			0.167			0.006
Urban	73 (61.9)	8561 (55.5)		1834 (53.5)	6800 (56.2)	
Rural	45 (38.1)	6858 (44.5)		1593 (46.5)	5310 (43.8)	
Antihypertensive medication use			<0.001			<0.001
Yes	7 (5.9)	3519 (22.8)		479 (14.0)	3047 (25.2)	
No	111(94.1)	11900 (77.2)		2948 (86.0)	9063 (74.8)	
Hypoglycemic agents use			0.533			<0.001
Yes	1 (0.8)	367 (2.4)		33 (1.0)	335 (2.8)	
No	117 (99.2)	15052 (97.6)		3394 (99.0)	11775 (97.2)	

Table 2 illustrates the basic and adjusted associations between blood pressure and cognitive impairment. In the crude model, the ORs (95% CIs) of cognitive impairment associated with hypotension, lower SBP and DBP were 3.12(2.17–4.49), 2.43(1.88–3.14), and 2.22(1.90–2.60), respectively. In the multivariable models, the adjusted ORs (95% CIs) were 1.62(0.97–2.69), 1.38(0.96–1.99), and 1.48(1.19–1.84), respectively. Compared with no-hypotension group, the ORs of cognitive impairment for isolated systolic hypotension, isolated diastolic hypotension, and sustained hypotension were 1.18(0.70–1.99), 1.45(1.14–1.84), and 1.66(1.00–2.76), respectively. The results of the sensitivity analysis with a cutoff score of 24 to define cognitive impairment were similar to this result (S1 Table).

**Table 2:** Association of blood pressure with cognitive impairment.

Blood pressure	N events/prevalence	Unadjusted model OR (95% CI)	Age-and sex-adjusted model OR (95% CI)	Multivariable-adjusted model^a^ OR (95% CI)
Hypotension (dichotomous)				
No	3372 (21.87%)	Reference	Reference	Reference
Yes	55 (46.61%)	3.12 (2.17–4.49)	2.58 (1.73–3.84)	1.62 (0.97–2.69)
Systolic pressure (mm Hg)				
>98	3327(21.76%)	Reference	Reference	Reference
≤98	100 (40.32%)	2.43 (1.88–3.14)	2.01 (1.52–2.67)	1.38 (0.96–1.99)
Diastolic pressure (mm Hg)				
>60	3160 (21.31%)	Reference	Reference	Reference
≤60	267 (37.55%)	2.22 (1.90–2.60)	1.63 (1.38–1.93)	1.48 (1.19–1.84)
Hypotension (multichotomous)^b^				
No-hypotension	3115 (21.2%)	Reference	Reference	Reference
Isolated systolic hypotension	45 (34.6%)	1.97 (1.37–2.83)	1.61 (1.08–2.40)	1.18 (0.70–1.99)
Isolated diastolic hypotension	212 (35.8%)	2.07 (1.74–2.46)	1.49 (1.24–1.80)	1.45 (1.14–1.84)
Sustained hypotension	55 (46.6%)	3.25 (2.26–4.67)	2.64 (1.77–3.94)	1.66 (1.00–2.76)

^a^ Multivariable-adjusted model including age, sex, education level, marital status, smoking status, drinking status, exercise, BMI, living arrangement, cardiovascular disease, diabetes, hypertension, functional disability, stroke, senile dementia, Parkinson’s diseases, registered residence, antihypertensive medication, hypoglycemic agents.

^b^ No-hypertension: SBP > 98 mm Hg and DBP > 60 mm Hg; Isolated systolic hypotension: SBP ≤ 98 mm Hg and DBP > 60 mm Hg; Isolated diastolic hypotension: SBP > 98 mm Hg and DBP ≤ 60 mm Hg; Sustained hypotension: SBP ≤ 98 mm Hg and DBP ≤ 60 mm Hg.

The basic and adjusted associations of blood pressure with CMMSE scores are shown in Table 3. In the multivariable models, CMMSE scores decreased by 2.08, 0.86, and 1.08 in the elderly with hypotension, lower SBP and DBP compared with those with non-hypotension, higher SBP and DBP, respectively. Compared with no-hypotension group, the βs of CMMSE scores for isolated systolic hypotension, isolated diastolic hypotension, and sustained hypotension were 0.23(-1.03–1.49), -0.87(-1.45- -0.29), and -2.12(-3.42- -0.82), respectively.

**Table 3:** Association of hypotension with CMMSE scores.

Blood pressure	Unadjusted model β (95% CI)	Age-and sex-adjusted model β (95% CI)	Multivariable-adjusted model^a^ β (95% CI)
Hypotension (no/yes)			
CMMSE scores	-5.74 (-7.33- -4.16)	-4.20 (-5.61- -2.80)	-2.08 (-3.38- -0.78)
Systolic pressure (no/yes)			
CMMSE scores	-4.14 (-5.24- -3.04)	-2.78 (-3.75- -1.80)	-0.86 (-1.78–0.05)
Diastolic pressure (no/yes)			
CMMSE scores	-3.71 (-4.37- -3.05)	-1.97 (-2.55- -1.38)	-1.08 (-1.61- -0.54)
Hypotension (multichotomous)^b^			
No-hypotension	Reference	Reference	Reference
Isolated systolic hypotension	-2.79 (-4.30- -1.29)	-1.53 (-2.87- -0.20)	0.23 (-1.03–1.49)
Isolated diastolic hypotension	-3.31 (-4.02- -2.59)	-1.53 (-2.16- -0.89)	-0.87 (-1.45- -0.29)
Sustained hypotension	-5.89 (-7.47- -4.31)	-4.28 (-5.69- -2.88)	-2.12 (-3.42- -0.82)

^a^ Multivariable-adjusted model including age, sex, education level, marital status, smoking status, drinking status, exercise, BMI, living arrangement, cardiovascular disease, diabetes, hypertension, functional disability, stroke, senile dementia, Parkinson’s diseases, registered residence, antihypertensive medication, hypoglycemic agents.

^b^ No-hypertension: SBP > 98 mm Hg and DBP > 60 mm Hg; Isolated systolic hypotension: SBP ≤ 98 mm Hg and DBP > 60 mm Hg; Isolated diastolic hypotension: SBP > 98 mm Hg and DBP ≤ 60 mm Hg; Sustained hypotension: SBP ≤ 98 mm Hg and DBP ≤ 60 mm Hg.

**Fig 2:**
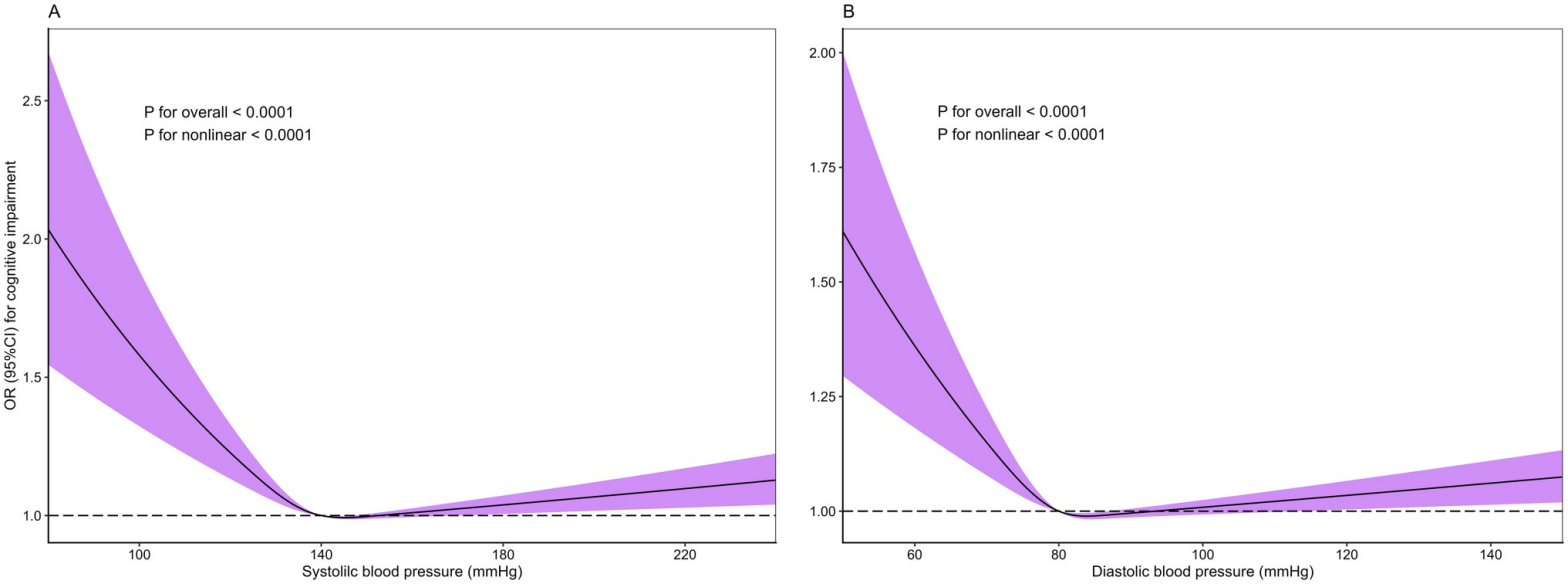
The dose-response relationships of SBP and DBP with cognitive impairment.

**Fig 3:**
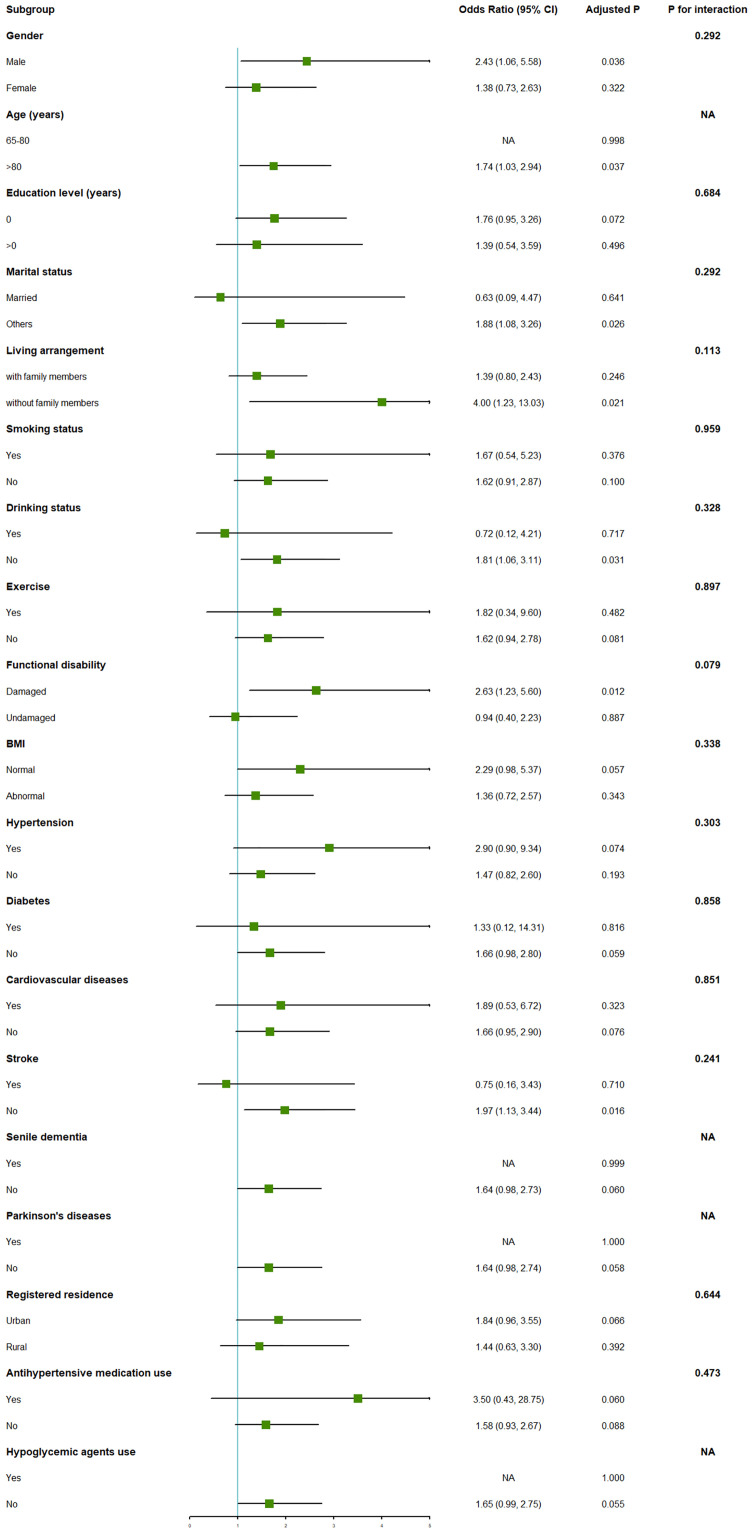
Subgroup analyses for cognitive impairment in the multivariable model.

The dose-response relationships of SBP and DBP with cognitive impairment are evaluated by RCS (Fig 2). The plots showed a near L-shaped association between SBP and DBP and cognitive impairment (*P*_nonlinear_ < 0.001 for SBP, and *P*_nonlinear_ = 0.001 for DBP). Fig 3 demonstrates the results of the subgroup analyses for cognitive impairment in the multivariable model. When compared to those with normal function of daily living, the elderly with decreased activity of daily living had higher OR of cognitive impairment associated with hypotension [2.63(1.23, 5.60) vs. 0.94 (0.40, 2.23), *P*_int_ = 0.079].

## Discussion

Based on CLHLS data, this study assessed the prevalence of hypotension and cognitive impairment in seniors of China, and the association between hypotension and cognitive function impairment. We found that hypotension was statistically significantly associated with cognitive impairment. The association was stronger in Chinese elderly with decreased activity of daily living. Moreover, there was statistical evidence of a nonlinear dose-response relationship of SBP and DBP with cognitive impairment. These findings may provide additional insight into the complex relationship between blood pressure and cognitive impairment.

In the current study, we also discovered that the prevalence of cognitive impairment screened by CMMSE was 22.06%, which seemed to be higher than that reported in other countries. The cognitive impairment prevalence was reported under 20% in most other countries, such as Portugal [27], UK [28, 29], and Brazil [30]. Therefore, more efficient and urgent prevention strategies for cognitive impairment should be provided to the Chinese elderly.

There have been inconsistent reports on the association between hypotension and cognitive impairment [31–35]. A nationally representative population-based survey in Malaysia revealed that hypotension (blood pressure <120/75 mm Hg) was significantly associated with decreased cognitive function (Beta = -0.11, *P* < 0.01), after adjusting for potential influencing factors [31]. Similarly, results from a study conducted by Guo et al. showed the elderly with SBP ≤ 140 mm Hg and DBP ≤ 75 mm Hg were more likely to suffer from demented than those with relatively higher SBP and DBP [32]. However, a study from Italy did not find a significant correlation between lower SBP (SBP < 120 mm Hg) and cognitive impairment in the elderly without heart failure [14]. Morris et al. found that DBP < 70 mm Hg as a potential risk factor for developing AD was not statistically significant [36]. Our study supports the notion that there is a potential increased risk of cognitive impairment with lower blood pressure. One reason for the inconsistency may stem from the variable criteria of hypotension utilized across countries and regions worldwide. Most literature indicates that an adult with a SBP < 80/90/110 mm Hg and/or a DBP < 60 mm Hg is considered hypotensive [37–39]. According to a large population-based survey in China, hypotension in adults is defined as SBP ≤ 98 mm Hg and DBP ≤ 60 mm Hg, which was the definition used in this study [21]. Another reason for the inconsistency is that the relationship between blood pressure and cognitive function may be altered by particular circumstances, such as age and baseline cognitive function. For example, the Leiden 85-plus Study found that lower systolic blood pressure in the oldest-old taking antihypertensives was associated with faster decline in cognitive function [40]. A population-based study in Japan showed that the relationship between SBP and cognitive function was closely related to baseline cognitive function [41]. To be specific, SBP was significantly and positively related to MMSE score in subjects with baseline MMSE < 24 points, whereas SBP was significantly and inversely related to MMSE score in subjects with MMSE ≥ 24 points. Therefore, interpretation of the data may require careful consideration of the actual value definition of low blood pressure and particular circumstances.

Additionally, in the current study, the prevalence of hypotension was 0.76% in the Chinese elderly, which was significantly lower than that reported in other countries. For instance, a study in Malaysia showed that the prevalence of hypotension was 29.3% among community-dwelling older adults [31]. Saks et al. reported that 11.1% of Estonians aged 65 years or older had hypotension [42]. The large variation in prevalence may also be due to different definitions of hypotension. We also found that the prevalence of cognitive impairment was significantly higher in women than in men (27.95% *vs*.14.46%). In fact, the association between sex and cognitive impairment is controversial [43–45]. Our findings supported the perception that women experienced cognitive impairment more frequently than men. This may be related to the reduced levels of estrogen in women’s bodies after menopause [46]. Another possible reason may be that women in our study had lower level of education relative to men, which has been shown to be a risk factor for cognitive impairment [47].

The results on the dose-response relationship of blood pressure with cognitive impairment were mixed. Some studies found linear, U-shaped, J-shaped, or no associations [10, 11, 48–51]. We found an L-shaped association between blood pressure and cognitive impairment, suggesting a strong adverse effect of low blood pressure on cognitive function. Notably, the current study applied RCS to visualize the complex relationship, which might provide further evidence for blood pressure and cognitive impairment. Furthermore, we discovered that the CMMSE scores decreased by 2.08 in the elderly with hypotension reaching the MCID score of 2 [19]. As the minimum threshold of improvement for patients, MCID has been widely used to indicate clinical practice implications. Therefore, preventing hypotension may be an effective approach to manage and control cognitive impairment in a clinical setting.

This study furthers our understanding of susceptible groups to the effects of hypotension. We discovered that the elderly with damaged function had higher odds ratios of cognitive impairment due to hypotension. The decreased activity of daily living may be an important sign of autonomic dysfunction [52], which can lead to poorer blood pressure regulation and greater susceptibility to cognitive impairment. Additionally, the elderly with impaired function in daily living are less physically active, whereas physical activity helps to mitigate the cognitive impairment associated with hypotension [53].

Although associations between hypotension and cognitive impairment are widely explored globally, the biological mechanisms still have not been fully clarified. The most credible perspective is that chronic hypotension may induce cerebral hypoperfusion, which in turn leads to cognitive dysfunction [54]. Researchers have suggested that a decline in blood pressure may reduce the velocity of cerebral blood flow and cause severe atherosclerosis. Evidence that cerebral hypoperfusion contributes to cognitive impairment is that reduced cerebral blood flow has been found in individuals with dementia and in those with early signs of dementia [55]. In addition, reduced cerebral blood flow in white matter resulting from chronic hypotension may progress to white matter lesions, which can lead to cognitive decline [56]. According to neuropathological studies, persistent cerebral hypoperfusion can also induce subcortical alterations [57]. However, a post hoc analysis of SPRINT MIND revealed that intensive blood pressure lowering appeared to be beneficial for mild cognitive impairment, especially for patients with low DBP [58]. There were also several other randomized controlled trials that showed no reduction or even an improvement in cerebral perfusion with intensive blood pressure control compared to standard blood pressure control [59, 60]. These findings seemed to suggest that lowering blood pressure or maintaining low blood pressure did not adversely affect cerebral blood flow and cognition. The accurate mechanisms underlying the deleterious effects of hypotension on cognitive impairment deserve further exploration.

There were several limitations that should be recognized. First, self-reported information of diseases may be susceptible to bias. The same pattern was also used in a previous study that focused on the prevalence and patterns of multimorbidity of the elderly, regardless of self-reported data [61]. If caregivers or relatives help the elderly to answer investigation questions, that may improve the quality of the investigation to an extent. Second, a few researchers indicated that the CMMSE test only partially reflected cognitive function of the elderly [62]. Third, the database did not collect the information on the use of cholinesterase inhibitors, a medication that may increase activity in the vagal nervous system and thus affect cognitive function. Finally, due to limited information in the database, we were unable to distinguish orthostatic hypotension, nocturnal hypotension, or intradialytic hypotension of the elderly, and therefore, we failed to further analyze the impacts of various hypotension on cognitive impairment.

## Conclusion

The current study provides evidence for the significant positive association between hypotension and cognitive impairment in Chinese elderly. The association was particularly stronger in seniors with reduced activity of daily living. Nonlinear dose-response relationships of SBP and DBP with cognitive impairment were observed. Our findings may provide additional epidemiological evidence for the effects of hypotension on cognitive impairment, and further studies are needed to examine the underlying biological mechanisms of hypotension related to cognitive impairment.

## Supporting information

S1 File Blood pressure measurements. (PDF)

S1 Table Association of blood pressure with cognitive impairment (CMMSE score < 24). (PDF)

## References

[1] Grover S. Aging population in Asia: Are we preparing ourselves enough? *Asian J Psychiatr* (2015) 13:1–2. doi: 10.1016/j.ajp.2015.02.003 25845323

[2] Feng Z, Zhan HJ, Feng X, Liu C, Sun M, Mor V. An industry in the making: the emergence of institutional elder care in urban china. *J Am Geriatr Soc* (2011) 59:738–44. doi: 10.1111/j.1532-5415.2011.03330.x 21410445 PMC3764455

[3] Bartels K, Esper SA, Thiele RH. Blood Pressure Monitoring for the Anesthesiologist: A Practical Review. *Anesth Analg* (2016) 122:1866–79. doi: 10.1213/ANE.0000000000001340 27195632

[4] Tadic M, Cuspidi C, Hering D. Hypertension and cognitive dysfunction in elderly: blood pressure management for this global burden. *BMC Cardiovasc Disord* (2016) 16:208. doi: 10.1186/s12872-016-0386-0 27809779 PMC5093934

[5] Santisteban MM, Iadecola C. Hypertension, dietary salt and cognitive impairment. *J Cereb Blood Flow Metab* (2018) 38:2112–2128. doi: 10.1177/0271678X18803374 30295560 PMC6282225

[6] McDonald C, Pearce MS, Kerr SRJ, Newton JL. Blood pressure variability and cognitive decline in older people: a 5-year longitudinal study. *J Hypertens* (2017) 35:140–147. doi: 10.1097/HJH.0000000000001120 27648719

[7] Posner HB, Tang M-X, Luchsinger J, Lantigua R, Stern Y, Mayeux R. The relationship of hypertension in the elderly to AD, vascular dementia, and cognitive function. *Neurology* (2002) 58:1175–81. doi: 10.1212/wnl.58.8.1175 11971083

[8] Levine DA, Galecki AT, Langa KM, Unverzagt FW, Kabeto MU, Giordani B, et al. Blood Pressure and Cognitive Decline Over 8 Years in Middle-Aged and Older Black and White Americans. *Hypertension* (2019) 73:310–318. doi: 10.1161/HYPERTENSIONAHA.118.12062 30624986 PMC6450556

[9] Lv Y-B, Zhu P-F, Yin Z-X, Kraus VB, Threapleton D, Chei C-L, et al. A U-shaped Association Between Blood Pressure and Cognitive Impairment in Chinese Elderly. *J Am Med Dir Assoc* (2017) 18:193.e7–193.e13. doi: 10.1016/j.jamda.2016.11.011 28126139 PMC5294228

[10] Molander L, Gustafson Y, Lövheim H. Longitudinal associations between blood pressure and dementia in the very old. *Dement Geriatr Cogn Disord* (2010) 30:269–76. doi: 10.1159/000320252 20847558

[11] Paran E, Anson O, Reuveni H. Blood pressure and cognitive functioning among independent elderly. *Am J Hypertens* (2003) 16:818–26. doi: 10.1016/s0895-7061(03)01005-7 14553960

[12] Skoog I, Lernfelt B, Landahl S, Palmertz B, Andreasson LA, Nilsson L, et al. 15-year longitudinal study of blood pressure and dementia. *Lancet* (1996) 347:1141–5. doi: 10.1016/s0140-6736(96)90608-x 8609748

[13] Schoon Y, Lagro J, Verhoeven Y, Rikkert MO, Claassen J. Hypotensive syndromes are not associated with cognitive impairment in geriatric patients. *Am J Alzheimers Dis Other Demen* (2013) 28:47–53. doi: 10.1177/1533317512466692 23242123 PMC10697226

[14] Zuccalà G, Onder G, Pedone C, Carosella L, Pahor M, Bernabei R, et al. Hypotension and cognitive impairment: Selective association in patients with heart failure. *Neurology* (2001) 57:1986–92. doi: 10.1212/wnl.57.11.1986 11739814

[15] Tang H-D, Zhou Y, Gao X, Liang L, Hou M-M, Qiao Y, et al. Prevalence and Risk Factor of Cognitive Impairment were Different between Urban and Rural Population: A Community-Based Study. *J Alzheimers Dis* (2016) 49:917–25. doi: 10.3233/JAD-150748 26519443

[16] Luzzi S, Vella L, Bartolini M, Provinciali L, Silvestrini M. Atherosclerosis in the evolution of Alzheimer’s disease: can treatment reduce cognitive decline? *J Alzheimers Dis* (2010) 20:893–901. doi: 10.3233/JAD-2010-091378 20182031

[17] *Chinese Longitudinal Healthy Longevity Survey (CLHLS) Community Datasets (1998–2018)* https://opendata.pku.edu.cn/dataverse/CHADS;jsessionid=121de1752184d9c4953cc0f28935 (Accessed August 13, 2023)

[18] Li H, Jia J, Yang Z. Mini-Mental State Examination in Elderly Chinese: A Population-Based Normative Study. *J Alzheimers Dis* (2016) 53:487–96. doi: 10.3233/JAD-160119 27163822

[19] Watt JA, Veroniki AA, Tricco AC, Straus SE. Using a distribution-based approach and systematic review methods to derive minimum clinically important differences. *BMC Med Res Methodol* (2021) 21:41. doi: 10.1186/s12874-021-01228-7 33637039 PMC7912575

[20] Nettleton D. Commercial Data Mining: Processing, Analysis and Modeling for Predictive Analytics Projects. Elsevier, 2014.

[21] Ju L, Zhao L, Yu W, Li S, Xu X, Fang H, et al. Prevalence and variation of hypotension in Chinese adult residents in 2002–2012. *Wei Sheng Yan Jiu* (2019) 48:869–875. 31875807

[22] Liu M, Committee of cardio-cerebro-vascular Disease of China Association of Gerontology and Geriatrics, Chinese College of Cardiovascular Physician of Chinese Medical Doctor Association. Chinese expert consensus on the diagnosis and treatment of hypertension in the elderly (2017). *Aging Med (Milton)* (2018) 1:106–116. doi: 10.1002/agm2.12020 31942486 PMC6880741

[23] Li C, Zhu Y, Ma Y, Hua R, Zhong B, Xie W. Association of Cumulative Blood Pressure With Cognitive Decline, Dementia, and Mortality. *J Am Coll Cardiol* (2022) 79(14):1321–1335. doi: 10.1016/j.jacc.2022.01.045 35393012

[24] Mathew A, Mesa RA, Nahodyl L, Tremblay J, Rundek T, Zeki Al Hazzouri A, et al. Diastolic Blood Pressure and Cognitive Functioning: Differences by Systolic Blood Pressure Among US Adults. *Am J Alzheimers Dis Other Demen* (2023) 38:15333175231172283. doi: 10.1177/15333175231172283 37177903 PMC10398835

[25] Durrleman S, Simon R. Flexible regression models with cubic splines. *Stat Med* (1989) 8:551–61. doi: 10.1002/sim.4780080504 2657958

[26] Ren Z, Li Y, Li X, Shi H, Zhao H, He M, et al. Associations of body mass index, waist circumference and waist-to-height ratio with cognitive impairment among Chinese older adults: Based on the CLHLS. *J Affect Disord* (2021) 295:463–470. doi: 10.1016/j.jad.2021.08.093 34507227

[27] Pais R, Ruano L, Moreira C, Carvalho OP, Barros H. Prevalence and incidence of cognitive impairment in an elder Portuguese population (65–85 years old). *BMC Geriatr* (2020) 20:470. doi: 10.1186/s12877-020-01863-7 33198643 PMC7667782

[28] Rait G, Fletcher A, Smeeth L, Brayne C, Stirling S, Nunes M, et al. Prevalence of cognitive impairment: results from the MRC trial of assessment and management of older people in the community. *Age Ageing* (2005) 34:242–8. doi: 10.1093/ageing/afi039 15863409

[29] Khanna AB, Metgud CS. Prevalence of cognitive impairment in elderly population residing in an urban area of Belagavi. *J Family Med Prim Care* (2020) 9:2699–2703. doi: 10.4103/jfmpc.jfmpc_240_20 32984110 PMC7491798

[30] Gondim AS, Coelho Filho JM, Cavalcanti A de A, de Roriz Filho J S, Nogueira CB, Peixoto AA Junior, et al. Prevalence of functional cognitive impairment and associated factors in Brazilian community-dwelling older adults. *Dement Neuropsychol* (2017) 11:32–39. doi: 10.1590/1980-57642016dn11-010006 29213491 PMC5619212

[31] Momtaz YA, Hamid TA, Haron SA, Bagat MF, Mohammadi F. Prevalence of hypotension and its association with cognitive function among older adults. *Aging Ment Health* (2018) 22:447–452. doi: 10.1080/13607863.2016.1268093 28060530

[32] Guo Z, Viitanen M, Fratiglioni L, Winblad B. Low blood pressure and dementia in elderly people: the Kungsholmen project. *BMJ* (1996) 312:805–8. doi: 10.1136/bmj.312.7034.805 8608286 PMC2350725

[33] Ou Y-N, Tan C-C, Shen X-N, Xu W, Hou X-H, Dong Q, et al. Blood Pressure and Risks of Cognitive Impairment and Dementia: A Systematic Review and Meta-Analysis of 209 Prospective Studies. *Hypertension* (2020) 76:217–225. doi: 10.1161/HYPERTENSIONAHA.120.14993 32450739

[34] Novak V, Hajjar I. The relationship between blood pressure and cognitive function. *Nat Rev Cardiol* (2010) 7:686–98. doi: 10.1038/nrcardio.2010.161 20978471 PMC3328310

[35] Sambati L, Calandra-Buonaura G, Poda R, Guaraldi P, Cortelli P. Orthostatic hypotension and cognitive impairment: a dangerous association? *Neurol Sci* (2014) 35:951–7. doi: 10.1007/s10072-014-1686-8 24590841

[36] Morris MC, Scherr PA, Hebert LE, Glynn RJ, Bennett DA, Evans DA. Association of incident Alzheimer disease and blood pressure measured from 13 years before to 2 years after diagnosis in a large community study. *Arch Neurol* (2001) 58:1640–6. doi: 10.1001/archneur.58.10.1640 11594923

[37] Mann A. Psychiatric symptoms and low blood pressure. *BMJ* (1992) 304:64–5. doi: 10.1136/bmj.304.6819.64 1737136 PMC1880984

[38] Critchley LA. Hypotension, subarachnoid block and the elderly patient. *Anaesthesia* (1996) 51:1139–43. doi: 10.1111/j.1365-2044.1996.tb15051.x 9038450

[39] Akahoshi M, Hida A, Imaizumi M, Soda M, Maeda R, Ichimaru S, et al. Basic characteristics of chronic hypotension cases: a longitudinal follow-up study from 1958 through 1999. *Hypertens Res* (2006) 29:1–7. doi: 10.1291/hypres.29.1 16715647

[40] Streit S, Poortvliet RKE, Gussekloo J. Lower blood pressure during antihypertensive treatment is associated with higher all-cause mortality and accelerated cognitive decline in the oldest-old. Data from the Leiden 85-plus Study. *Age Ageing* (2018) 47(4):545–550. doi: 10.1093/ageing/afy072 29741555

[41] Ishikawa J, Seino S, Kitamura A, Toba A, Toyoshima K, Tamura Y, et al. The relationship between blood pressure and cognitive function. *Int J Cardiol Cardiovasc Risk Prev* (2021) 10:200104. doi: 10.1016/j.ijcrp.2021.200104 35112116 PMC8790103

[42] Saks K, Kolk H, Soots A, Takker U, Vask M. Prevalence of cardiovascular disorders among the elderly in primary care in Estonia. *Scand J Prim Health Care* (2003) 21:106–9. doi: 10.1080/02813430310001716 12877374

[43] Langa KM, Levine DA. The diagnosis and management of mild cognitive impairment: a clinical review. *JAMA* (2014) 312(23):2551–61. doi: 10.1001/jama.2014.13806 25514304 PMC4269302

[44] Li W, Sun L, Xiao S. Prevalence, Incidence, Influence Factors, and Cognitive Characteristics of Amnestic Mild Cognitive Impairment Among Older Adult: A 1-Year Follow-Up Study in China. *Front Psychiatry* (2020) 11:75. doi: 10.3389/fpsyt.2020.00075 32184742 PMC7058542

[45] Liu Y, Yu X, Han P, Chen X, Wang F, Lian X, et al. Gender-specific prevalence and risk factors of mild cognitive impairment among older adults in Chongming, Shanghai, China. *Front Aging Neurosci* (2022) 14:900523. doi: 10.3389/fnagi.2022.900523 36118698 PMC9475287

[46] Russell JK, Jones CK, Newhouse PA. The Role of Estrogen in Brain and Cognitive Aging. *Neurotherapeutics* (2019) 16(3):649–665. doi: 10.1007/s13311-019-00766-9 31364065 PMC6694379

[47] Sattler C, Toro P, Schönknecht P, Schröder J. Cognitive activity, education and socioeconomic status as preventive factors for mild cognitive impairment and Alzheimer’s disease. *Psychiatry Res* (2012) 196(1):90–5. doi: 10.1016/j.psychres.2011.11.012 22390831

[48] Morris MC, Scherr PA, Hebert LE, Bennett DA, Wilson RS, Glynn RJ, et al. Association between blood pressure and cognitive function in a biracial community population of older persons. *Neuroepidemiology* (2002) 21:123–30. doi: 10.1159/000054809 12006775

[49] Gottesman RF, Schneider ALC, Albert M, Alonso A, Bandeen-Roche K, Coker L, et al. Midlife hypertension and 20-year cognitive change: the atherosclerosis risk in communities neurocognitive study. *JAMA Neurol* (2014) 71:1218–27. doi: 10.1001/jamaneurol.2014.1646 25090106 PMC4226067

[50] Waldstein SR, Giggey PP, Thayer JF, Zonderman AB. Nonlinear relations of blood pressure to cognitive function: the Baltimore Longitudinal Study of Aging. *Hypertension* (2005) 45:374–9. doi: 10.1161/01.HYP.0000156744.44218.74 15699446

[51] Thorvaldsson V, Skoog I, Hofer SM, Börjesson-Hanson A, Ostling S, Sacuiu S, et al. Nonlinear blood pressure effects on cognition in old age: separating between-person and within-person associations. *Psychol Aging* (2012) 27:375–83. doi: 10.1037/a0025631 21988152 PMC3625422

[52] Merola A, Romagnolo A, Rosso M, Suri R, Berndt Z, Maule S, et al. Autonomic dysfunction in Parkinson’s disease: A prospective cohort study. *Mov Disord* (2018) 33(3):391–397. doi: 10.1002/mds.27268 29278286

[53] Angevaren M, Aufdemkampe G, Verhaar HJ, Aleman A, Vanhees L. Physical activity and enhanced fitness to improve cognitive function in older people without known cognitive impairment. *Cochrane Database Syst Rev* (2008) (3):CD005381. doi: 10.1002/14651858.CD005381.pub3 18646126

[54] Kalback W, Esh C, Castaño EM, Rahman A, Kokjohn T, Luehrs DC, et al. Atherosclerosis, vascular amyloidosis and brain hypoperfusion in the pathogenesis of sporadic Alzheimer’s disease. *Neurol Res* (2004) 26:525–39. doi: 10.1179/016164104225017668 15265270

[55] Ruitenberg A, den Heijer T, Bakker SLM, van Swieten JC, Koudstaal PJ, Hofman A, et al. Cerebral hypoperfusion and clinical onset of dementia: the Rotterdam Study. *Ann Neurol* (2005) 57:789–94. doi: 10.1002/ana.20493 15929050

[56] Mehrabian S, Duron E, Labouree F, Rollot F, Bune A, Traykov L, et al. Relationship between orthostatic hypotension and cognitive impairment in the elderly. *J Neurol Sci* (2010) 299:45–8. doi: 10.1016/j.jns.2010.08.056 20855089

[57] Brun A, Englund E. A white matter disorder in dementia of the Alzheimer type: a pathoanatomical study. *Ann Neurol* (1986) 19:253–62. doi: 10.1002/ana.410190306 3963770

[58] Jiang C, Li S, Wang Y, Lai Y, Bai Y, Zhao M, et al. Diastolic Blood Pressure and Intensive Blood Pressure Control on Cognitive Outcomes: Insights From the SPRINT MIND Trial. *Hypertension* (2023) 80(3):580–589. doi: 10.1161/HYPERTENSIONAHA.122.20112 36688305

[59] Tryambake D, He J, Firbank MJ, O’Brien JT, Blamire AM, Ford GA. Intensive blood pressure lowering increases cerebral blood flow in older subjects with hypertension. *Hypertension* (2013) 61(6):1309–15. doi: 10.1161/HYPERTENSIONAHA.112.200972 23529166

[60] Croall ID, Tozer DJ, Moynihan B, Khan U, O’Brien JT, Morris RG, et al. Effect of Standard vs Intensive Blood Pressure Control on Cerebral Blood Flow in Small Vessel Disease: The PRESERVE Randomized Clinical Trial. *JAMA Neurol* (2018) 75(6):720–727. doi: 10.1001/jamaneurol.2017.5153 29507944 PMC5885221

[61] Yao S-S, Cao G-Y, Han L, Chen Z-S, Huang Z-T, Gong P, et al. Prevalence and Patterns of Multimorbidity in a Nationally Representative Sample of Older Chinese: Results From the China Health and Retirement Longitudinal Study. *J Gerontol A Biol Sci Med Sci* (2020) 75:1974–1980. doi: 10.1093/gerona/glz185 31406983

[62] Gutierrez J, Marshall RS, Lazar RM. Indirect measures of arterial stiffness and cognitive performance in individuals without traditional vascular risk factors or disease. *JAMA Neurol* (2015) 72:309–15. doi: 10.1001/jamaneurol.2014.3873 25599130 PMC8985655

