# Supplementary material for: Hypotension and cognitive impairment among the elderly: Evidence from the CLHLS
Source: PLoS One. 2023 Sep 19;18(9):e0291775. doi: 10.1371/journal.pone.0291775 (PMC10508618; doi:10.1371/journal.pone.0291775)
Supplement: S1 File — (PDF) [file pone.0291775.s001.pdf]

S1 File. Blood pressure measurements.

Requirements for calibrating mercury sphygmomanometers

(1) The amount of mercury in the sphygmomanometer must be sufficient. After turning on the sphygmomanometer, the mercury convex surface in the graduated tube should be exactly at scale 0, and the air hole at the upper end of the glass tube cannot be blocked.

(2) The air in the airbag before measurement is completely evacuated, and there must be no air leakage in the airbag.

Preparations for blood pressure measurement

(1) Participants need to take off thick clothes, expose the left upper arm, or wear only thin clothing.

(2) The subject sits on a chair with his feet flat on the ground and his arms on the table.

(3) The investigator wraps the cuff approximately 1-2 cm above the left upper arm's elbow socket, with the arrow under the air tube at the center of the inside of the arm.

(4) The investigator secures the cuff (the cuff should be in direct contact with the subject's skin as much as possible). The subject should be able to easily insert his index finger between the cuff and the arm.

(5) The subject’s arm is placed on the table with the palms facing up, and the center of the cuff is kept at the same height as the heart.

(6) If the subject cannot remain in the sitting position, the arm is placed by the bed when measuring in the supine position. If when the supine position, the upper arm is below the heart level, then a pillow can be placed under the upper arm.

(7) During the measurement, the investigator should try to not let the subject see the display of the sphygmomanometer.

(8) The interviewer needs to take the blood pressure of the elderly individuals twice, with an interval of at least 1 minute.
