# Supplementary material for: Hypotension and cognitive impairment among the elderly: Evidence from the CLHLS
Source: PLoS One. 2023 Sep 19;18(9):e0291775. doi: 10.1371/journal.pone.0291775 (PMC10508618; doi:10.1371/journal.pone.0291775)
Supplement: S1 Table — (PDF) [file pone.0291775.s002.pdf]

S1 Table. Association of blood pressure with cognitive impairment (CMMSE score < 24).

Blood pressure	N events/prevalence	Unadjusted model OR (95% CI)	Age-and sex-adjusted model OR (95% CI)	Multivariable-adjusted model[a] OR (95% CI)
Hypotension (dichotomous)				
No	5455 (35.38%)	Reference	Reference	Reference
Yes	76 (64.41%)	3.31 (2.26- 4.82)	2.82 (1.84- 4.31)	1.82 (1.06- 3.11)
Systolic pressure (mm Hg)				
>98	5395(35.29%)	Reference	Reference	Reference
≤98	136 (54.84%)	2.23 (1.73- 2.87)	1.83 (1.38- 2.43)	1.26 (0.88- 1.80)
Diastolic pressure (mm Hg)				
>60	5155 (34.77%)	Reference	Reference	Reference
≤60	376 (52.88%)	2.11 (1.81- 2.45)	1.52 (1.29- 1.79)	1.27 (1.03- 1.56)
Hypotension (multichotomous)[b]				
No-hypotension	5095 (34.67%)	Reference	Reference	Reference
Isolated systolic hypotension	60 (46.15%)	1.62 (1.14- 2.28)	1.26 (0.85- 1.85)	0.91 (0.55- 1.50)
Isolated diastolic hypotension	300 (50.59%)	1.93 (1.64- 2.27)	1.36 (1.13- 1.62)	1.18 (0.94- 1.49)
Sustained hypotension	76 (64.41%)	3.41 (2.34- 4.98)	2.86 (1.87- 4.38)	1.83 (1.07- 3.13)

[a] Multivariable-adjusted model including age, sex, education level, marital status, smoking status, drinking status, exercise, BMI, living arrangement, cardiovascular disease, diabetes, hypertension, functional disability, stroke, senile dementia, Parkinson's diseases, registered residence, antihypertensive medication, hypoglycemic agents.

[b] No-hypertension: SBP > 98 mm Hg and DBP > 60 mm Hg; Isolated systolic hypotension: SBP ≤ 98 mm Hg and DBP > 60 mm Hg; Isolated diastolic hypotension: SBP > 98 mm Hg and DBP ≤ 60 mm Hg; Sustained hypotension: SBP ≤ 98 mm Hg and DBP ≤ 60 mm Hg.
